# The mechanism behind lack-of-effect of lanthanum on seed germination of switchgrass

**DOI:** 10.1371/journal.pone.0212674

**Published:** 2019-03-04

**Authors:** He Xueqing

**Affiliations:** College of Grassland Agriculture, Northwest A&F University, Yangling, Shaanxi Province, China; College of Agricultural Sciences, UNITED STATES

## Abstract

Switchgrass (*Panicum virgatum* L.) is a perennial warm-season C_4_ grass identified as a model species for bioenergy feedstock production. Lanthanum (La) as a rare earth element can stimulate the physiological processes of plant growth. The purpose of this study was to investigate the effect of lanthanum on seed germination of switchgrass. However, no significant differences in seed germination were found. The energy dispersive X-ray analysis showed that abundant lanthanum deposits resided on the pericarp and testa of the seed while few lanthanum deposits were present on the aleurone and endosperm. This phenomenon demonstrates that a semi-permeable layer, which could restrict or impede solute exchange, while allowing the permeability of internal and external water and gas, may be located between the testa and aleurone. Light microscopy and histochemical analysis revealed that the main chemical composition of the semi-permeable layer would be expected to be suberin because the layer was stained yellow with aniline blue. The quantum chemical calculations predict that the intervals between adjacent carbon chains in suberin molecule are so small that lanthanum ([La(H_2_O)_8_]^3+^) cannot pass through the suberin molecule. In conclusion, the seed germination of switchgrass is not affected by lanthanum because the semi-permeable layer restricts the penetration of lanthanum into the embryo.

## Introduction

Switchgrass (*Panicum virgatum* L.) is a perennial warm-season C_4_ grass identified as a model species for bioenergy feedstock production [1] [2]. Switchgrass has been used as a ground cover, forage for livestock, soil and water conservation, and wildlife habitat. The U.S. Department of Energy (DOE) recommended switchgrass as a model herbaceous biofuel crop, as switchgrass produces high yield, contains high levels of cellulose, requires low energy input for production, grows in marginal lands, and can be a dedicated biofuel crop [3]. Overall, it is considered a resource-efficient, low-input crop for producing bioenergy from farmland.

Rare earth elements (REE) are a homogenous group of 17 chemical elements in the periodic table that are key to many modern industries, such as those which produce chemicals, consumer electronics, clean energy, transportation, medical usage, aviation and defense [4]. Moreover, they have been commonly used as microfertilizers in agriculture since the 1980s, due to their capabilities to enhance seed germination, root growth, chlorophyll content, plant resistance to abiotic stress, and productivity of some crops at low concentrations [5] [6] [7]. Numerous studies have demonstrated that REEs have an important effect on plant growth and development [8] [9] [10]. Lanthanum is one of the rare earth elements, which has been widely used to produce fluorescent materials and fertilizer additives [11] [12]. A large number studies have shown that lanthanum has an effect on seed germination [13] [14] [15] [16]. However, the sensitivity of switchgrass seed germination to lanthanum and the mechanism by which lanthanum affects the seed germination of switchgrass are unknown. The objectives of this research are to evaluate the effect of lanthanum on seed germination of switchgrass, and to reveal the underlying mechanism of lanthanum’s effect.

## Materials and methods

### Seed material

The mature switchgrass seeds of the lowland cultivar Alamo were harvested during October 2016 from the Experiment Station of Grassland Science in Yangling (N34°16′, E 108°4′), Shaanxi Province, PR China. Seeds were cleaned and stored in paper bags at 4°C until used. According to the International Seed Testing Rules [17], thousand-seed weight was1.921 g, or the initial moisture content was 8.7%.

### Seed germination test

Seeds were surface sterilized with 75% alcohol for 30 s, rinsed with sterile water and then seeds were treated in two ways: soaking and wetting. For the soaking treatments, seeds were soaked in 0.01 mM, 0.1 mM, 1 mM and 10 mM La(NO_3_)_3_ solution in a water carrier at 20°Cfor 24 h in the dark, while control seeds were treated with an equal amount of distilled water, and dried on blotting paper. Seeds were left overnight at room temperature. After drying, the moisture content of soaked seeds was similar to the initial moisture content, as determined by comparing the total seed weights. For the wetting treatment, seeds were wetted with 0.01 mM, 0.1 mM, 1 mM and 10 mM La(NO_3_)_3_ solutions. The germination test was conducted with 250 seeds (five replicates groups, and each group contains 50 seeds) for each treatment. Seeds were sown in an 9 cm diameter Petri dishes with 2 layers of filter paper saturated with 6 mL water or solutions. The dishes were placed in an incubator for 21 d. During the germination test, the temperature was maintained at 25±2°C, 60% humidity on a16/8 h light/dark cycle, to 75 μmol m^-2^ s^-1^ of irradiance, which was given during the high temperature period each day [18]. Water was added to the dishes as necessary during the test period. The location of dishes in the incubator was changed randomly each day. The criterion for germination was observance of the visible radicle protrusion (≥ 2 mm) [19].

### Energy dispersive X-ray (EDX) analysis

The palea and lemma were removed from 25 seeds. The ‘naked’ seeds were soaked in 10 mM La(NO_3_)_3_ at 20°Cfor 24 h in the dark, and then removed from solution. Finally, they were aired at room temperature. Seeds were cut longitudinally with a razor blade and mounted on a metal sample table. The seed structure was viewed under a scanning electron microscope (JSM 5600 LV, Japan) equipped with an energy dispersive X-ray spectrometer (KEVE2, America). According to the features of the lanthanum spectrum, we located the position of the semi-permeable layer.

### Light microscopy and histochemical analysis

The ‘naked’ seeds (without palea and lemma) in deionized water were placed in a growth chamber at 20°C for 24 h. The seeds with surface breakage or visible radicles were discarded, and the surfaces of the remaining seeds were cut with a razor blade to remove approximately 1 mm^3^ of the caryopsis coat, along with a small amount of endosperm tissue. The extracted samples were fixed in 3% glutaraldehyde solution at 4°C for 24 h. The fixed samples were rinsed with phosphate buffer saline (PBS, 0.1 M, pH 7.2) five times for 10 min each time. Then the materials were fixed again with 1% osmic acid at 4°C for 1.5 h. The rinsing process was the same as before. Then samples were dehydrated by different concentrations of alcohol, once with 30%, 50%, 70%, 80% and 90% for 20 min each time and twice with 100% for 30 min each time. The segments were dehydrated again twice with 100% acetone for 30 min each time. Following alcohol dehydration, the samples were embedded specimen in epoxy resin: acetone and epoxy resin at a ratio of 3:1 for 2 h; acetone and epoxy resin at a ratio of 1:1 for 4 h; acetone and epoxy resin at a ratio of 1:3 for 12 h; pure epoxy resin then drying at 30°C for 24 h; and pure epoxy resin then drying at 60°C for 48 h. Next, the specimen were cut into slices 1 μm thick with leica semi-automatic rotary slicer (RM 2245, Germany) and were stained with 0.05% aniline blue to determine their microscopic anatomical structures and chemical composition. In the last step, the sections were mounted with Canadian balasm and were observed and photographed under a optical microscope.

### Quantum chemical calculations

Suberin is the main chemical composition of suberized cell walls. On the basis of the suberin macromolecular structure proposed by Graca and Santos [20], quantum chemical calculations were carried out to investigate the stable structure of suberin and the binding interaction of suberin with La^3+^. It should be mentioned that La^3+^ is mainly in the form of [La(H_2_O)_8_]^3+^ [21] [22] in the present solution (pH≤6.5). The quantum chemical calculations were performed by using the Gaussian 09 program package (Gaussian Inc.) [23]. The structure of suberin is optimized by the semi-empirical parametric method 3 (PM3) method, and the geometries of [La(H_2_O)_8_]^3+^ and suberin-[La(H_2_O)_8_]^3+^ complexes are optimized at the density functional theory with Becke-3-Lee-Yang-Parr functional (DFT-B3LYP) level [24] with a mix basis set, i.e., the 6-31G (d) basis set for C, H and O atoms, and Stuttgart–Dresden (SDD) basis set for La^3+^ atom.

### Statistical analysis

Experimental data were analyzed with SPSS 19.0 software. Means were compared by one-way analysis of variance(ANOVA), and Duncan’s test was used for multiple comparisons of the different treatments.

## Results

### Effect of La(NO_3_)_3_ on seed germination of switchgrass

**Fig 1:**
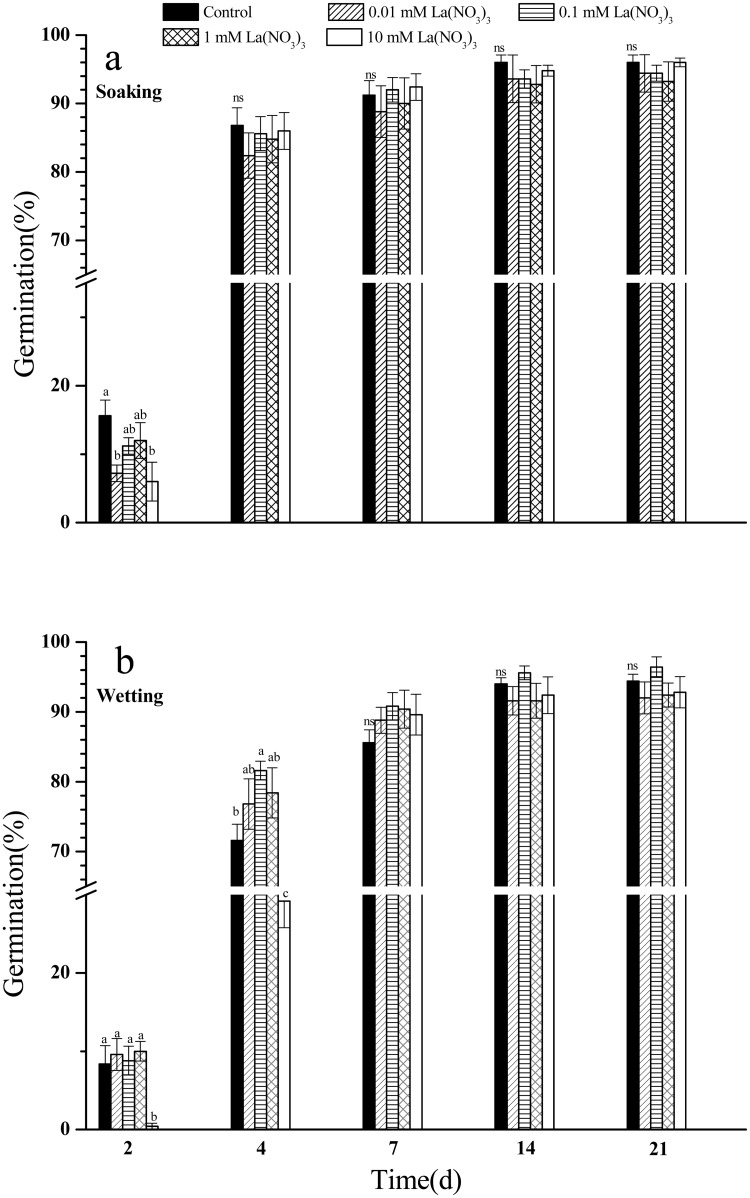
Seed germination of switchgrass with soaking and wetting in La(NO_3_)_3_. Values represent the mean (±SE) of five independent experiments; different letters indicates that the values are significantly different from controls (*P*<0.05). ns stands for no significant for the case that all treatments have the same letter.

Soaking treatments with different concentrations of La(NO_3_)_3_ reduced the seed germination of switchgrass at day 2 compared to the control (Fig 1a, S1 Table), while they had no significant on seed germination at and after day 4. Wetting treatment with the highest concentration of lanthanum inhibited the seed germination at day 2 and 4 in comparison to the control (Fig 1b, S2 Table). However, wetting treatments with all tested concentrations of lanthanum did not induce significant variations in seed germination at and after day 7. Overall, lanthanum with different concentration gradients had no significant effect on seed germination both under soaking and wetting treatments (*P*>0.05) at and after day 7.

### Energy dispersive X-ray (EDX) analysis

**Fig 2:**
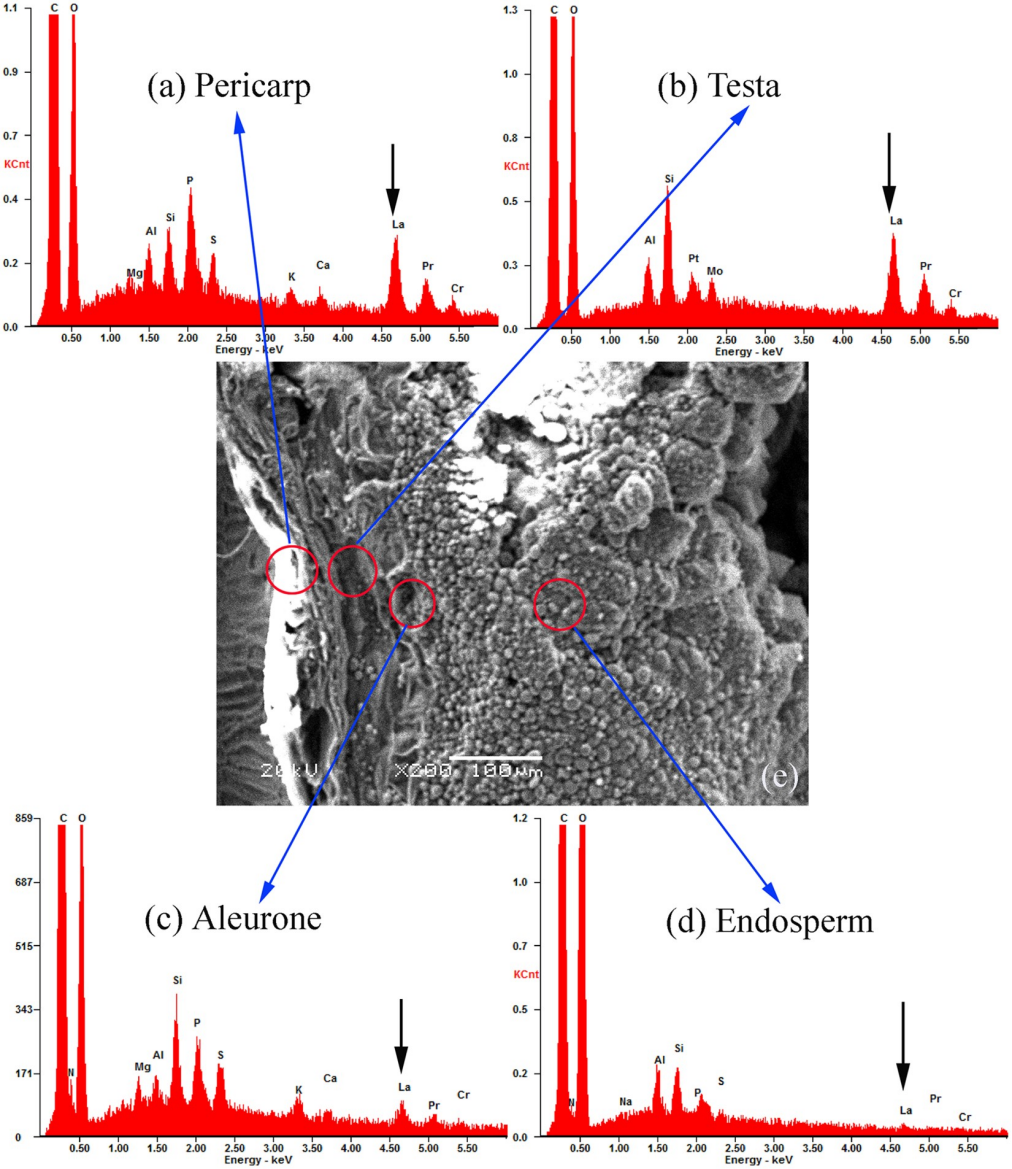
Energy dispersive X-ray images of the seed coat. The pericarp (a), testa (b), aleurone (c), endosperm (d) and scanning electron microscope images of seed (e).

The position of the semi-permeable layer was presumed to be the location where the lanthanum was prevented from penetrating further into the seed. During analysis, attention was focused on the location of lanthanum deposits. Abundant lanthanum deposits were found on the pericarp (shown by the black arrow) (Fig 2a), and its content was 0.24% (atomic percent) or 2.48% (Weight) (Table 1). Analogously, abundant lanthanum deposits were located on the testa (Fig 2b), and its content was increased from 0.24% to 0.47% (atomic percent). Detailed analyses revealed that few lanthanum deposits were present in the aleurone layer (Fig 2c) and endosperm (Fig 2d), which indicated that the lanthanum had not infiltrated into the aleurone and endosperm. The absence of a lanthanum peak suggests that the semi-permeable layer of the switchgrass seeds was located at the innermost layer of the testa and directly adjacent to the aleurone.

**Table 1:** Comparison of mineral element distributions in seed of the switchgrass.

Position	Content(%)										
		C	N	O	Mg	Al	Si	P	S	K	La
**Pericarp**	Atom^a^(%)	78.44	0	20.25	0.07	0.20	0.25	0.37	0.12	0.04	0.24
	Weight(%)	70.78	0	24.34	0.14	0.41	0.53	0.86	0.28	0.10	2.48
**Testa**	Atom(%)	68.01	0	29.89	0	0.47	0.97	0	0	0	0.47
	Weight(%)	57.21	0	33.49	0	0.89	1.91	0	0	0	4.55
**Aleurone**	Atom(%)	67.19	6.94	24.48	0.22	0.15	0.42	0.28	0.17	0.05	0.07
	Weight(%)	60.06	7.23	29.15	0.41	0.30	0.89	0.65	0.41	0.15	0.70
**Endosperm**	Atom(%)	56.18	2.73	40.34	0	0.31	0.24	0.07	0.02	0	0.01
	Weight(%)	48.89	2.77	46.77	0	0.60	0.50	0.17	0.04	0	0.10

^a^Atom represents atomic percent.

### Seed coat structure and chemical composition of semi-permeable layer of switchgrass

**Fig 3:**
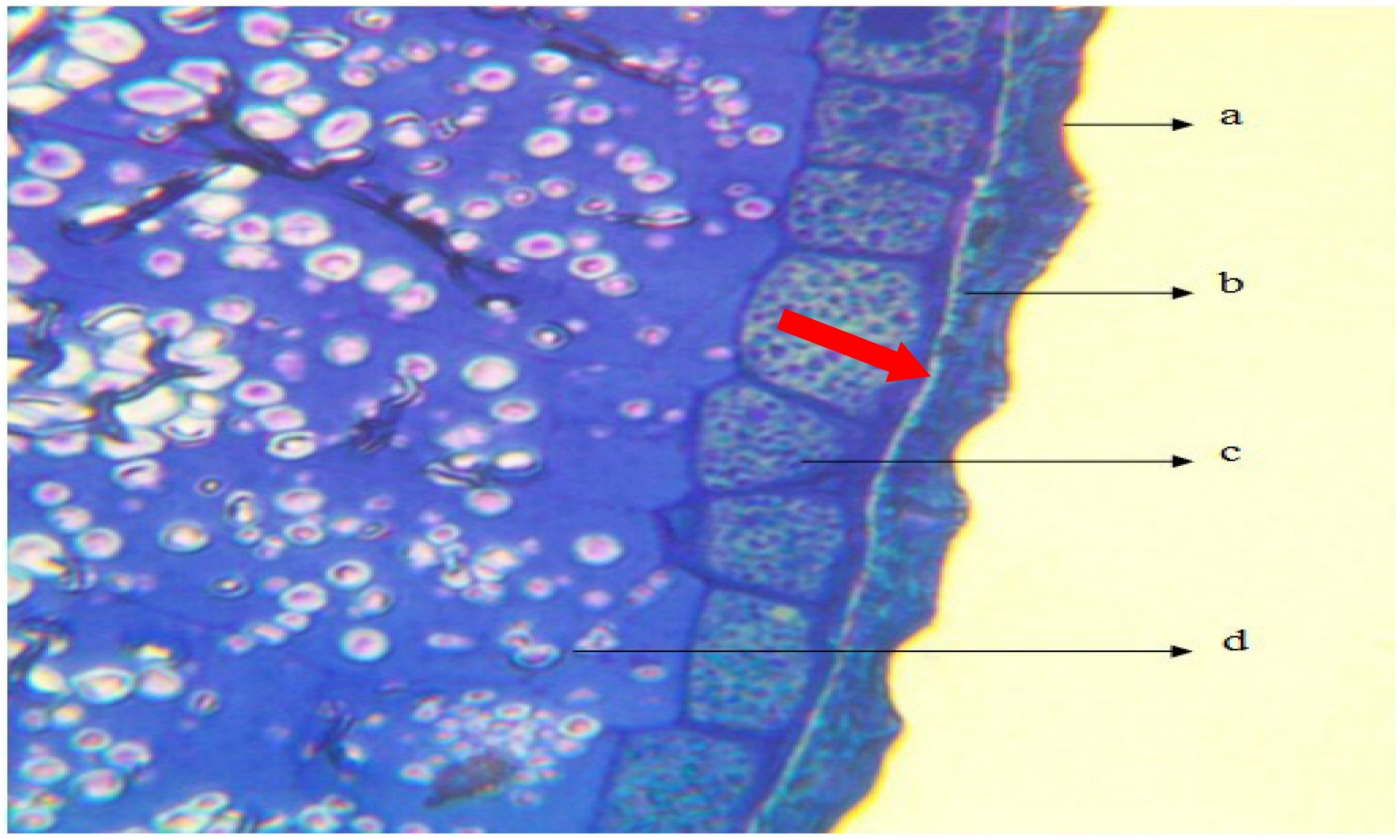
Seed coat anatomical structure of switchgrass. a: pericarp; b: testa; c: taleurone; d: endosperm.

The main structure of the seed includes the pericarp (a), testa (b), aleurone (c) and endosperm (d) (Fig 3). Based on observations, there was a thin layer of tissue (shown by the red arrow) that has been stained yellow located inside the testa and close to the aleurone layer. It was determined to be a semi-permeable layer, and the main chemical composition was determined to be suberin.

### The stable structure of suberin and the binding interaction of suberin with La^3+^

**Fig 4:**
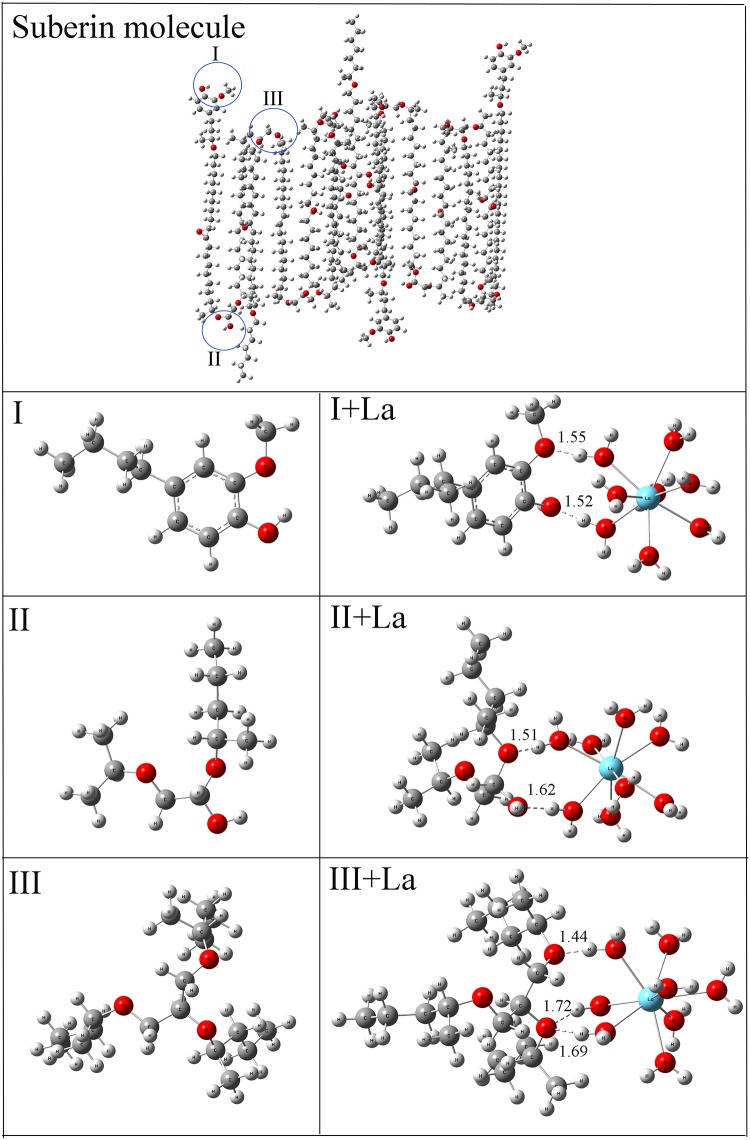
The quantum chemical results of the optimized suberin molecule and the interactions between suberin and [La(H_2_O)_8_]^3+^. I, II and III stand for three kinds of binding sites in suberin molecule. I: The binding site with one phenolic-OH and one phenolic-OCH3. II: The binding site with one C-OH and one C-O-C group. III: The binding site with two C-O-C groups. The dashed lines between O and H atoms in I+La, II+La and III+La represent H-bonds, and the numbers above the dashed lines are bond lengths of H-bonds.

The optimized geometry of suberin molecule has a layered structure (Fig 4), and the intervals between adjacent carbon chains are so small that [La(H_2_O)_8_]^3+^ cannot penetrate suberin molecule. Moreover, if suberin molecules are stacked one by one in the suberized semi-permeable layer, [La(H_2_O)_8_]^3+^ would not pass through it, which is consistent with our experimental observation. There are many binding sites in the suberin molecule for La(H_2_O)_8_^3+^, but these binding sites can be divided into three kinds. The first kind of binding site (I) is composed of one phenolic-OH and one phenolic-OCH_3_ group. The second one (II) consists of one C-OH and one C-O-C group. The third one is made up of two C-O-C groups. The calculated results show that La(H_2_O)_8_^3+^ is anchored to these binding sites by H-bonds. The hydrogen atoms in La(H_2_O)_8_^3+^ could form H-bonds with the O atoms at the binding sites, and the lengths of the H-bonds range between 1.44 and 1.72 Å. Our calculations also predict that the binding energies (E(suberin-[La(H_2_O)_8_]^3+^ complex)—E(suberin)—E(La(H_2_O)_8_^3+^)) are -268.85, -297.47 and -375.71 kJ mol^-1^ for the first, second and third kind of binding sites, respectively, which demonstrates that the suberin-[La(H_2_O)_8_]^3+^ complexes are very stable. From Fig 4, it is seen that there are always 2 or 3 H-bonds between the suberin and [La(H_2_O)_8_]^3+^, and these phenomena explain why the suberin-[La(H_2_O)_8_]^3+^ complexes are stable although the polarities of the binding sites are weak.

## Discussion

Lanthanum has an hormesis effect which refers to the stimulation of physiological processes of an organism by poisoning substances, on plant growth and development [25]. However, its effect varies across different plant species. In a study on *Triticum durum*, seed pre-soaking for 8 h with lanthanum inhibited seed germination at low concentrations (0.01 mM and 0.1 mM), while pre-soaking for only 2 and 4 h inhibited seed germination when higher concentrations (1 mM and 10 mM) of lanthanum were used [8]. Another study reported that lanthanum promoted the seed germination of *Lycospersicon esukurentamu* at a concentration of 0.01 mM, while high concentration (10 mM) had exactly the opposite effect [26]. Our present investigations show that soaking or wetting the seed of switchgrass with 10 mM La(NO_3_)_3_ inhibits the seed germination at day 2, which is due to the fact that the high concentration of lanthanum may reduce the water imbibition of seed at the beginning of seed germination test. However, lanthanum could not affect the seed germination after day 7. This interesting phenomenon stimulates us to design further experiments to reveal the underlying mechanism.

The semi-permeable layer, which could restrict or impede solute exchange while allowing the permeability of internal and external water and gas, exists in many seed coats, and its location varies with the different plant species. The semi-permeable layer of *Elymus nutans* and *E*. *sibiricus* is located at the outermost layer of the seed coat and is connected to the pericarp [27]. The semipermeable layer is firmly attached on the external portion of the seed coat in *Roegneria nutans*, *Achnatherum inebrians*, *Hordeum vulgare* var. *nudum*, *Festuca sinensis*, and *Bromus inermis* seeds [28]. Studies on *Sorghum sudanense* seeds show that the semipermeable layer is located on the inner side of the aleurone layer, close to the undifferentiated cells, and is, hence, endospermic [29]. For *Lolium perenne*, the semipermeable layer is located at the surface of the testa cells [30]. In *Hordeum vulgare* [31], *Triticum aestivum* [32], *Secale cereal* [33] and *Zea mays* seed [34], it is found that the semipermeable layer was within the inner or outer part of the seed coat. *Lactuca sativa* seeds have an endospermic semi-permeable layer around the embryo [35]. For switchgrass, Salanenka and Taylor [36] found that the structure of seed prevented the penetration of fluorescent dye beyond of the seed coat, meaning the semi-permeable layer of the plant is located in the seed coat. Our EDX analysis shows that abundant lanthanum deposits reside on the pericarp and testa while few lanthanum deposits are present on the aleurone layer and endosperm, which indicates that the semi-permeable layer of switchgrass is located at the innermost layer of the testa and is firmly attached to the aleurone layer.

Previous studies have suggested that the semipermeable layer of seeds mainly consists of cellulose, cutin, suberin, lipid and callose [27] [37] [38]. Grace and Santos [20] further reported that suberin was represented up to 50% of the chemical composition of the cell wall. The present study found that the semi-permeable layer of switchgrass was stained yellow with aniline blue [39], which demonstrated that its main chemical composition should be suberin. Quantum chemical calculations can provide information at the molecular level, and many studies [40] demonstrate that metal ions can be bound to chemical components of seed by coordinate covalent bonds or H-bonds. Based upon the suberin macromolecular structure proposed by Graca and Santos [20], our quantum chemical calculations suggest that the suberin molecule had a layered structure, and [La(H_2_O)_8_]^3+^ could not penetrate it because the intervals between adjacent carbon chains were very small. Our finding further explained and confirmed the conclusion that the semi-permeable layer is an important structure for restricting the penetration of toxic solutes into embryos during imbibition from the soil [41].

## Conclusions

In this study, the seed of switchgrass is determined to have a semi-permeable layer, which is located between testa and aleurone layer, and the main chemical composition of the semi-permeable layer is suberin. The suberin molecule restricts the penetration of La^3+^ (in the form of La(H_2_O)_8_^3+^) into embryo, which explains the lack of effect on seed germination of switchgrass by lanthanum.

## Supporting information

S1 Table Effect of La(NO_3_)_3_ on seed germination of switchgrass by soaking. (PDF)

S2 Table Effect of La(NO_3_)_3_ on seed germination of switchgrass by wetting. (PDF)
