# Supplementary material for: The mechanism behind lack-of-effect of lanthanum on seed germination of switchgrass
Source: PLoS One. 2019 Mar 4;14(3):e0212674. doi: 10.1371/journal.pone.0212674 (PMC6398849; doi:10.1371/journal.pone.0212674)
Supplement: S1 Table — (PDF) [file pone.0212674.s001.pdf]

S1 Table. Effect of $La(NO_3)_3$ on seed germination of switchgrass by soaking.

Soaking Treatment	Germination(%)				
	2 d	4 d	7 d	14 d	21 d
Control-1	12	86	88	96	96
Control-2	10	86	90	94	94
Control-3	14	86	94	96	96
Control-4	22	80	86	94	94
Control-5	20	96	98	100	100
0.01 mM-1	6	88	92	98	98
0.01 mM-2	6	88	90	98	98
0.01 mM-3	10	70	74	80	84
0.01 mM-4	10	84	94	98	98
0.01 mM-5	4	82	94	94	94
0.1 mM-1	10	84	94	94	96
0.1 mM-2	10	88	96	98	98
0.1 mM-3	14	94	94	94	94
0.1 mM-4	8	82	86	90	92
0.1 mM-5	14	80	90	92	92
1 mM-1	6	80	86	90	90
1 mM-2	8	88	94	96	98
1 mM-3	20	96	100	100	100
1 mM-4	10	76	78	84	84
1 mM-5	16	84	92	94	94
10 mM-1	0	92	96	96	98
10 mM-2	8	92	94	96	96
10 mM-3	16	80	86	92	96
10 mM-4	4	80	90	94	94
10 mM-5	2	86	96	96	96
