# Supplementary material for: The mechanism behind lack-of-effect of lanthanum on seed germination of switchgrass
Source: PLoS One. 2019 Mar 4;14(3):e0212674. doi: 10.1371/journal.pone.0212674 (PMC6398849; doi:10.1371/journal.pone.0212674)
Supplement: S2 Table — (PDF) [file pone.0212674.s002.pdf]

S2 Table. Effect of $La(NO_3)_3$ on seed germination of switchgrass by wetting.

Wetting Treatment	Germination(%)				
	2 d	4 d	7 d	14 d	21 d
Control-1	10	66	82	92	92
Control-2	8	68	86	92	92
Control-3	16	78	92	96	96
Control-4	6	70	82	96	96
Control-5	2	76	86	94	96
0.01 mM-1	2	88	92	92	92
0.01 mM-2	10	80	88	92	92
0.01 mM-3	10	76	92	96	98
0.01 mM-4	14	74	90	94	94
0.01 mM-5	12	66	82	84	84
0.1 mM-1	14	78	92	98	100
0.1 mM-2	6	82	84	94	94
0.1 mM-3	8	86	92	94	94
0.1 mM-4	12	82	90	94	94
0.1 mM-5	4	80	96	98	100
1 mM-1	10	74	92	94	94
1 mM-2	14	74	92	94	94
1 mM-3	10	70	80	82	86
1 mM-4	10	88	92	92	92
1 mM-5	6	86	96	96	96
10 mM-1	2	34	92	94	94
10 mM-2	0	36	92	96	96
10 mM-3	0	34	92	94	94
10 mM-4	0	22	94	96	96
10 mM-5	0	20	78	82	84
